# Anal squamous cell carcinoma in a high HIV prevalence population

**DOI:** 10.1007/s12672-021-00397-7

**Published:** 2021-02-11

**Authors:** Danielle R. L. Brogden, Christopher C. Khoo, Christos Kontovounisios, Gianluca Pellino, Irene Chong, Diana Tait, Oliver J. Warren, Mark Bower, Paris Tekkis, Sarah. C. Mills

**Affiliations:** 1 Chelsea and Westminster Hospitals NHS Foundation Trust, London, UK; 2 Imperial College London, London, UK; 3 Royal Marsden NHS Foundation Trust, London, UK; 4 Institute of Cancer Research, London, UK; 5 Department of Advanced Medical and Surgical Sciences, Università Degli Studi Della Campania “Luigi Vanvitelli”, Naples, Italy; 6 Colorectal Surgery, Vall d’Hebron University Hospital, Barcelona, Spain

**Keywords:** Anal squamous cell carcinoma, HIV, Chemoradiotherapy

## Abstract

Anal Squamous Cell Carcinoma (ASCC) is a rare cancer that has a rapidly increasing incidence in areas with highly developed economies. ASCC is strongly associated with HIV and there appears to be increasing numbers of younger male persons living with HIV (PLWH) diagnosed with ASCC. This is a retrospective cohort study of HIV positive and HIV negative patients diagnosed with primary ASCC between January 2000 and January 2020 in a demographic group with high prevalence rates of HIV. One Hundred and seventy six patients were included, and clinical data was retrieved from multiple, prospective databases. A clinical subgroup was identified in this cohort of younger HIV positive males who were more likely to have had a prior diagnosis of Anal Intraepithelial Neoplasia (AIN). Gender and HIV status had no effect on staging or disease-free survival. PLWH were more likely to develop a recurrence (p < 0.000) but had a longer time to recurrence than HIV negative patients, however this was not statistically significant (46.1 months vs. 17.5 months; p = 0.077). Patients known to have a previous diagnosis of AIN were more likely to have earlier staging and local tumour excision. Five-year Disease-Free Survival was associated with tumour size and the absence of nodal or metastatic disease (p < 0.000).

## Introduction

Anal Squamous Cell Carcinoma (ASCC) is considered a rare cancer, with an incidence rate that is frequently quoted as between 1 and 2 cases per 100,000 per year worldwide [1]. Nevertheless, the incidence of ASCC is rapidly increasing; Islami et al. [2] demonstrated a percentage incidence increase of between 2.8 and 6.8% per year in men and 1.5–10% per year in woman in Western developed countries between 1971 and 2007.

The known risk factors for the development of ASCC include immunosuppression, smoking, receptive anal intercourse, sexually transmitted infections, previous or current Human Papillomavirus (HPV) related dysplasia and malignancies and Human Immunodeficiency Virus (HIV) [3].

HIV is a significant risk factor in the development of ASCC, and ASCC is the third most common cancer in people living with HIV (PLWH) [4]. It is likely that that the increasing incidence of ASCC related to the higher number of PLWH in developed countries where, in the era of Highly Active Antiretroviral Therapy (HAART), PLWH are able to achieve a near normal life expectancy and go on to develop long-term sequelae of HIV infection [5]. Despite improving outcomes of HIV prognosis with HAART, the use of HAART does not reduce the risk of ASCC in PLWH [4], therefore it is likely that the incidence of ASCC in PLWH will continue to rise [6].

ASCC is associated with persistent oncogenic Human Papillomavirus (HPV) infections and has a known HPV related dysplastic precursor; Anal Intraepithelial Neoplasia (AIN). With a similar natural history to Cervical Intraepithelial Neoplasia (CIN), it hypothesised that like cervical cancer, ASCC could become a largely preventable cancer with HPV vaccination, ASCC screening programmes and appropriate treatment for AIN when identified.

Nevertheless, the screening for, and treatment of, AIN remains controversial in clinical guidelines [7, 8]. Although clinical guidelines identify PLWH as a high-risk group for ASCC, there is significant discrepancy in the literature regarding the progression rates of high-grade AIN to ASCC [9–12] and, unlike cervical cancer screening programmes which have a good evidence base in preventing malignancy, corresponding ASCC screening programmes have not been shown to be as effective. There is also a lack of evidence that treating AIN once it has been identified can prevent ASCC [13].

Despite the controversy, many HIV centres provide local screening services for PLWH. In our clinical centre, a high resolution anoscopy and cytology screening programme is offered to PLWH and in particular men who have receptive anal intercourse (MSM).

Historically, the majority of ASCC diagnosed are in women in their 6^th^ or 7^th^ decade who are likely to have had a previous high-risk HPV infection and the published literature focuses mainly on this patient demographic [8, 14, 15]. Although, research involving the screening and treatment of AIN is centred around the newly increasing PLWH demographic, there is less clinical evidence about the outcomes of the PLWH subgroup once they are diagnosed with ASCC.

We present our experience with managing patients with ASCC over two decades between 2000 to 2020 with emphasis on whether there exists a separate subpopulation of ASCC patients with HIV within the Trust and whether this affects clinical prognosis.

## Methods

We undertook a retrospective cohort study following the “strengthening the reporting of observational studies in epidemiology (STROBE)” statement [16]. Consecutive patients undergoing treatment for Anal Squamous Cell Carcinoma between January 2000 and January 2020 were identified from prospectively maintained clinical databases. The departments of sexual health, surgery and HIV kept separate clinical databases across two clinical sites. Data entries were linked by hospital number and date of birth and then anonymised for analysis. The study protocol was reviewed and approved by the London-Westminster Research and Ethics Committee prior to data collection.

### Inclusion criteria

The study included all adult patients over 18 years of age diagnosed with primary histological proven Anal Squamous Cell Carcinoma (ASCC) between January 2000 and January 2020.

### Exclusion criteria

Patients under 18 years of age. Anal cancers with a histopathology other than Squamous Cell Carcinoma were excluded from this analysis.

### Data collection

For each patient included in the study, the authors retrieved data including demographics, HIV outcomes, attendance at screening, histopathology, staging, and treatment received (Table 1) from separate clinical databases and combined it to form one data record per patient. Staging and treatment were commonly repeated across clinical databases, and any discrepancies were independently checked by two independent clinical reviewers (DRLB and CCK).

**Table 1:** Extracted data for analysis

Demographics	Age	Sex	Co-morbidities	Performance status
HIV	Date of Diagnosis	AIDS Diagnosis	Viral Load	CD4 count
Sexual Health	Hepatitis B	Hepatitis C	Attendance at screening	Previous AIN Diagnosis
Pathology	Staging	Tumour differentiation	Surgical Treatment	Previous HPV related cancers
Oncological Treatment	Chemotherapy	Radiotherapy	Last date of follow up	Response to treatment

### Outcome measures

The outcome measures were recurrence, time to recurrence and 5-year disease free survival in PLWH and HIV-negative patients.

### Statistical analysis

Data were analysed using SPSS Statistics software. A statistically significant p value was defined as p < 0.05 for this analysis. Chi squared tests and Fishers exact tests were used for categorical variables and ANOVA tests were used for comparisons between means.

## Results

### Patient characteristics

**Fig. 1:**
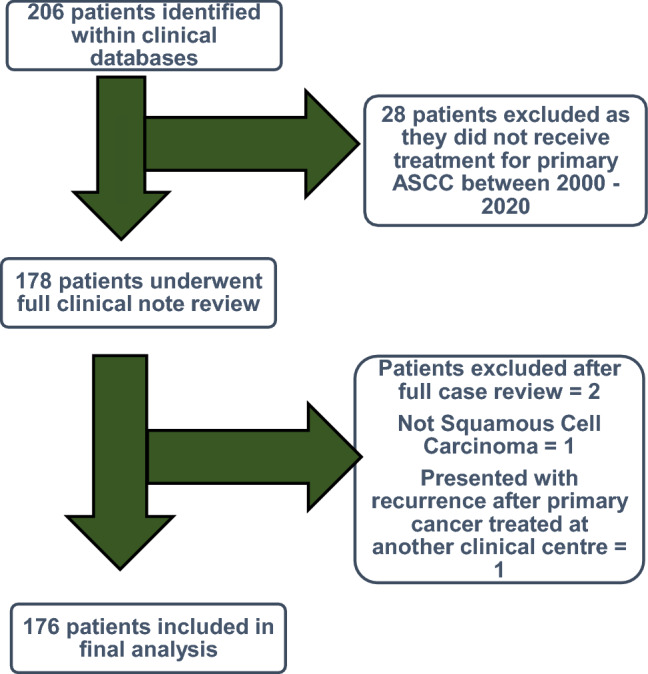
STROBE flow chart. Flow chart describing method of identification of patients included in cohort and reasons for exclusion

One hundred and seventy six patients were included (see Fig. 1 for STROBE flow diagram), 55 patients (31%) were female, the mean age at diagnosis was 56.5 years (median 53 years, range 31–92 years) (See Table 2 and 3 for study demographics). Ninety six patients were HIV positive (55%) and 32 patients (18%) were HIV negative. 27% of patients did not have a documented HIV status on retrospective chart review, they were statistically more likely to be female (p < 0.001, Chi Squared) and have a previous diagnosis of Genitourinary Intraepithelial Neoplasia (p = 0.011, Fisher’s Exact Test).

**Table 2:** Patient demographics and staging

Demographics	Male (n = 121)	Female (n = 55)	p-value
Mean age (years)	53.2 Standard Deviation = 13.4 Range 31–92	62.8 Standard Deviation = 13.1 Range 33–90	p < 0.001
HIV status	Positive 92 (76%), Negative 13 (11%) Not tested 16 (13%)	Positive 4 (7%), Negative 19 (35%) Not tested 32 (58%)	p < 0.001
Previous AIN	43 (34%)	2 (4%)	p < 0.001
Previous GIN	1 (1%)	8 (15%)	p < 0.001
Stage 1	47 (39%)	15 (27%)	P = 0.191
stage 2A	17 (14%)	10 (18%)	
Stage 2B	4 (3%)	1 (2%)	
Stage 3A	7 (6%)	5 (9%)	
Stage 3B	13 (11%)	2 (4%)	
Stage 3C	19 (18%)	13 (24%)	
Stage 4	8 (7%)	2 (4%)

**Table 3:** Patient demographics by documented HIV status

Demographics	HIV Positive (n = 96)	HIV Negative (n = 32)	Not Tested (n = 48)	p-value
Mean age (years)	47.9 Standard Deviation = 8.6 Range 31–73 years	64.1 Standard Deviation = 14.2 Range 32–90 years	66.0 Standard Deviation = 12.5 Range 38–92	P < 0.001
Previous AIN	39 (41%)	3 (9%)	3 (6%)	p < 0.001
Previous GIN	1 (1%)	3 (10%)	5 (10%)	p = 0.011
Stage 1	40 (42%)	11 (34%)	11 (23%)	P = 0.071
Stage 2A	13 (14%)	5 (16%)	9 (19%)	
Stage 2B	4 (4%)	1 (3%)	0 (0%)	
Stage 3A	7 (7%)	1 (3%)	4 (8%)	
Stage 3B	11 (12%)	1 (3%)	3(6%)	
Stage 3C	16 (17%)	6 (19%)	10 (21%)	
Stage 4	3 (17%)	4 (13%)	3 (6%)	

Of the patients with a known HIV status, 75% were PLWH in this cohort. 76% of male patients were PLWH whereas only 7% of female patients were PLWH (p < 0.001, Chi Squared). 38% of PLWH included in this cohort had previously met the criteria for AIDS during their treatment for HIV. 38% of PLWH had a concurrent diagnosis of Hepatitis B, 22% Hepatitis C and 10% had both Hepatitis B and C.

### Risk factors

#### Age

Men presented with ASCC at an earlier age than women (53.2 years compared to 62.8 years; p < 0.001, ANOVA). PLWH were also statistically more likely to present at younger age when compared to HIV negative patients (47.9 years compared to 64.1 years; p < 0.001, ANOVA). There was no statistical difference in age when comparing patients with and without a previous AIDs diagnosis, previous diagnosis of intraepithelial neoplasia, other predisposing skin conditions and immunosuppression. Patients with metastatic disease were statistically more likely to be older (67.5 years compared to 54.3 years; p = 0.007, ANOVA). With the exception of patients receiving Radiotherapy alone who were statistically older than patients receiving other treatment modalities (78.3 years compared to 54.5 years; p < 0.001, ANOVA) there was no statistical difference in age and choice of treatment modality between groups.

#### Gender

**Fig. 2:**
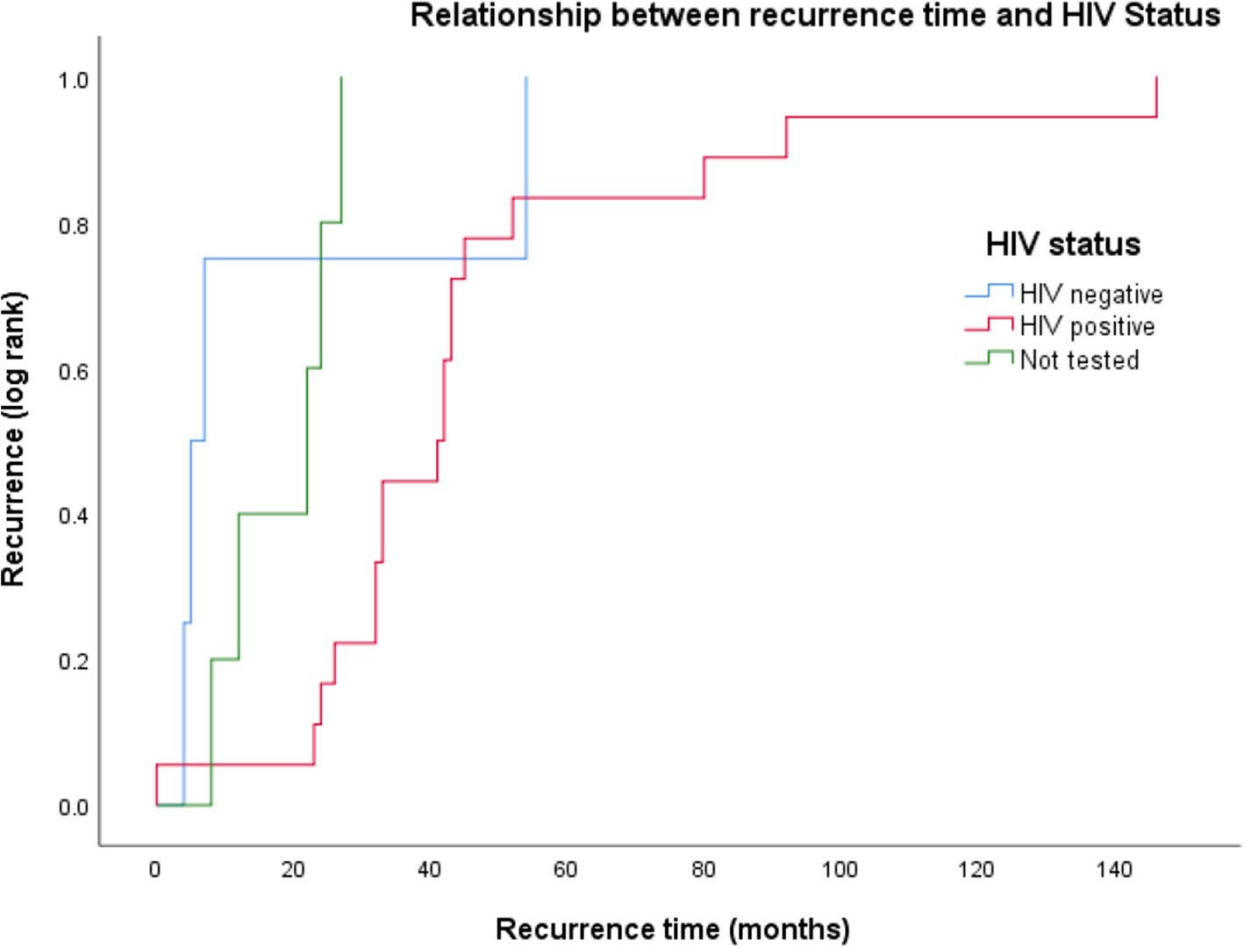
The relationship between HIV status and recurrence time. Log rank Kaplan Meier Curve demonstrating time from primary ASCC diagnosis to development of local or distant recurrent disease stratified by HIV test status

Men were more likely to have HIV (p < 0.001, Chi Squared), and to have had previous treatment for AIN (p < 0.001, Fisher’s Exact Test). A higher proportion of male patients presented with an early cancer (Stage 1 and 2: 56% compared to 47%) but this was not statistically significant (Fig. 2). There was no difference in gender proportions when comparing treatment modalities, recurrence rates and disease-free survival.

#### Intraepithelial Neoplasia and other predisposing skin conditions

Three patients had predisposing anal skin conditions linked to ASCC such as lichen sclerosis and only 3% of patients had a pre-disposing co-morbidity leading to immunosuppression other than HIV status.

Four patients had concurrent cervical and or vulval intraepithelial neoplasia and three patients had a prior treated vulval squamous cell carcinoma. One patient with high-grade cervical intraepithelial neoplasia was also HIV positive. 26% were previously diagnosed with AIN and had received follow up and/or treatment before ASCC diagnosis.

Having a previous known diagnosis of AIN before ASCC diagnosis was associated with being HIV positive (p < 0.001, Fisher’s Exact Test), but there was not a similar effect with patients diagnosed with other non-anal genitourinary intraepithelial neoplasia. Being known to have AIN was associated with PLWH presenting with a stage 1 cancer when compared to PLWH without a previous AIN diagnosis (p < 0.001, Fisher’s Exact Test). As a result, they were also more likely to have local excision as treatment (p < 0.001, Fisher’s Exact Test) and less likely to have chemoradiotherapy.

### TMN staging and treatment of ASCC

50% of patients presented with early-stage disease (Stage 1 35%; Stage 2A 15%) and 5% of patients presented with metastatic disease.

42% had their tumour locally excised, 61% had Chemoradiotherapy and 4% had Radiotherapy alone. 14% had a defunctioning colostomy for symptomatic control and 5% eventually underwent an abdominoperineal excision for recurrent disease. PLWH were more likely to receive chemoradiotherapy (p = 0.009, Fisher’s Exact Test) and less likely to have palliative radiotherapy alone (p < 0.001, Fisher’s Exact Test) (Table 4).

**Table 4:** Treatment received by HIV status

Treatment received	HIV positive (n = 96)	HIV Negative (n = 32)	p-value
Chemoradiotherapy	63 (66%)	19 (59%)	p = 0.009
Palliative radiotherapy only	1 (1%)	3 (9%)	p < 0.000
Local excision of tumour	30 (31%)	19 (59%)	p = 0.07
Abdominoperineal resection	2 (2%)	3 (9%)	p = 0.140
Defunctioning stoma	16 (17%)	4 (13%)	p = 0.419
Local excision of tumour only	24 (25%)	6 (19%)	p = 0.016

### Clinical outcomes

#### Recurrence

19% (n = 33) of patients developed a recurrence; 30 patients after completing chemoradiotherapy. 7 patients underwent an Abdominoperineal Resection as salvage surgery after their recurrence, no immediate post-operative complications were identified. There was no statistical association between recurrence rate and age, sex, hepatitis status, previous AIN treatment or previous cervical or vulval dysplasia. PLWH were more likely to develop a recurrence compared to HIV negative and non-tested patients (Table 5). However, PLWH also took longer to develop recurrent disease when compared to HIV negative patients (46.1 months compared to 17.5 months) but this was not statistically significant; (p = 0.077, ANOVA) (Fig. 2). As they are demographically very similar (see Table 3) if we group untested and HIV negative patients together this statistic becomes significant (46.1 months vs. 18.1 months; p = 0.022, ANOVA).

**Table 5:** Recurrence and disease-free survival in HIV positive and HIV negative patients

End outcomes	HIV positive (n = 96)	HIV negative (n = 32)	p-value
Recurrence	22 (23%)	4 (13%)	P < 0.001
5-year disease free survival	49 (51%)	9 (28%)	p = 0.181

PLWH that recurred were more likely to have received chemoradiotherapy (p = 0.031, Fisher’s Exact Test) and have a moderately or poorly differentiated malignancy (Table 6).

**Table 6:** Recurrence variable analysis

HIV status	HIV positive		HIV negative		p-value
Demographics	Recurrence (N = 22)	No Recurrence (N = 74)	Recurrence (N = 4)	No Recurrence (N = 28)	(Fishers Exact Test)
Gender	Male 19 (86%) Female 3 (14%)	Male 73 (99%) Female 1 (1%)	2 (50%) 2 (50%)	11 (39%) 17 (61%)	P = 0.037
Previous AIN	8 (36%)	31 (42%)	1 (25%)	2 (7%)	P = 0.780
Previous GIN	1 (5%)	0 (0%)	0 (0%)	3 (11%)	P = 0.229
Previous AIDs criteria	9 (41%)	27 (37%)	n/a	n/a	P = 0.763
Hepatitis status HBV HCV HBV and HCV	9 (41%) 5 (23%) 3 (14%)	23 (31%) 14 (19%) 6 (8%)	0 (0%) 0 (0%) 0 (0%)	2 (7%) 2(7%) 1 (4%)	P = 0.775 P = 0.927 P = 0.927
Staging 1 2A 2B 3A 3B 3C 4 Not staged	7 (32%) 3 (14%) 4 (18%) 2 (9%) 3 (14%) 7 (32%) 0 (0%) 0 (0%)	33 (45%) 10 (14%) 4 (5%) 5 (7%) 8 (11%) 9 (12%) 3 (4%) 2 (3%)	1 (25%) 2 (50%) 0 (0%) 0 (0%) 0 (0%) 0 (0%) 0 (0%) 1 (25%)	10 (36%) 3 (11%) 1 (4%) 1 (4%) 1 (4%) 6 (21%) 4 (14%) 2 (7%)	P = 0.443 P = 0.433 P = 0.750 P = 0.750 P = 0.067 P = 0.600
Chemoradiotherapy	19 (89%)	44 (60%)	3 (75%)	16 (57%)	P = 0.031
Local excision of tumour	4 (18%)	26 (35%)	2 (50%)	17 (60%)	P = 0.104
Tumour differentiation Foci of SCC Well differentiated Moderately differentiated Poorly differentiated	1 (5%) 2 (9%) 12 (55%) 3 (14%)	7 (10%) 9 (12%) 29 (39%) 7 (10%)	0 (0) 0 (0) 2 (50%) 1 (25%)	4 (14%) 1 (4%) 8 (29%) 6 (21%)	P = 1.000 P = 1.000 P = 0.028 P = 0.286

### Disease free survival

23% of patients died of ASCC during routine 5-year follow-up. Overall, 42% of patients achieved 5-year disease free survival and 9% were lost to follow up. 26% were diagnosed within the last 5 years and therefore could not be included in the survival analysis.

Survival was associated with < 5 cm tumour size (p < 0.000, Chi Squared) and the absence of nodal or metastatic disease at presentation (p < 0.000, Chi Squared). 70% of patients achieving 5-year Disease Free Survival were T1/2 compared to only 27% of patients with T3/T4 tumours. No patients with distant metastatic disease on presentation achieved 5-year Disease Free survival.

There was no statistical association between disease free survival and HIV status, gender, and previous AIN or non-anal intraepithelial dysplasias and malignancies.

50% of patients who were given a stoma for symptomatic control were eventually reversed. 33% patients died with a stoma in situ.

## Discussion

ASCC is a cancer of increasing incidence in the western world [2], which may in part be due to the increasing prevalence of HIV and the increasing incidence of ASCC in PLWH treated with HAART [6, 17]. This cohort study was undertaken to examine patients with ASCC at a specialist HIV referral centre with emphasis on whether PLWH have different clinical outcomes to their HIV negative counterparts.

Within this cohort, we identified a clinical subgroup of younger male PLWH who were more likely to have a previous diagnosis of AIN. The cohort also showed a difference in recurrence rates and HIV status, although HIV negative patients on average recurred earlier than PLWH there was no difference in mortality. Taking this into consideration, it is possible that as PLWH are statistically younger at the time of ASCC diagnosis, that PLWH with good viral control have a longer expected life span when compared to older HIV negative patients and are therefore they more likely to develop a recurrence in later life. Our data suggests that long term follow up, beyond the routine 5 years after ASCC treatment, may be advisable for some PLWH as they are more likely to recur later than patients without a positive HIV test (p = 0.022, ANOVA).

The use of High Resolution Anoscopy (HRA) as a screening tool remains controversial. In recent clinical guidelines, The Association of Coloproctology in Great Britain and Ireland could not recommend screening practices such as HRA for routine clinical practice [8]. Similarly, the American Society of Colon and Rectal Surgeons limited its recommendations for the use of HRA to experienced practitioners performing screening on high risk individuals only [7]. These recommendations are based on a lack of evidence that HRA is a screening method that prevents cancer. A recent systematic review also did not identify any evidence based treatments that could definitely prevent the onset of ASCC if high-grade AIN were detected [13]. As yet there are very few studies with long enough follow up times to be able to identify a risk reduction.

Interestingly, PLWH within our cohort who also had a prior diagnosis of AIN were more likely to present with early-stage tumours (< 5 cm). We believe that this could be an important finding as the earlier detection of disease is an important criterion for a successful screening programme. Due to the retrospective nature of this cohort study, we cannot be certain however whether this finding is a result of cancer being detected earlier by HRA or whether a selection bias exists as patients who comply with a voluntary screening programme are more likely to be informed about the risks of ASCC and present earlier. There was no associated survival benefit identified but this is unsurprising as unfortunately the size of the cohort is underpowered to do so. Nevertheless, there is likely to be a significant benefit to quality of life as patients with smaller tumours are less likely to recur or require permanent colostomy. Only larger datasets involving PLWH screening programmes will be able to identify any benefits with certainty.

Recently, there have been encouraging developments in establishing datasets evaluating AIN management and ASCC prevention. The Multinational Anal Squamous Cell Carcinoma: Audit and Registry (mASCARA) started recruiting patients in 2019 [3], and pursues a collaborative approach combining sexual health, gynaecology, HIV and oncology outcomes. The dataset is being used to investigate the screening, prevention, and early diagnosis of ASCC. Moreover, the Anal Cancer HSIL Outcomes Research (ANCHOR) study (NCT02135419) is a multicentre randomised controlled trial based in the USA, which aims to determine whether the treatment of AIN does prevent ASCC. The use of datasets could improve patient outcomes and open the era of adaptive therapy for cancer patients, ideally suiting multicentric registries and international collaboratives [3, 18, 19].

### Strengths and limitations of study

This is a large cohort of patients with a rare cancer, in a specialist centre for the management of HIV and ASCC. The data available permitted the review of a 20-year experience of treating ASCC in a demographic where HIV is highly prevalent. There is little previous data comparing the outcomes between HIV positive and HIV negative patient groups.

Notwithstanding the large cohort for a rare cancer; we are limited by recall bias and a high number of patients being lost to follow up. The data collection for PLWH was also more detailed in the first few years of the cohort when compared to HIV negative patients.

Interestingly, our experience did not identify any relationship between Hepatitis B and C infections that have been shown to be a risk of ASCC in other studies [20, 21]. and unfortunately the data available did not permit an analysis based on previous anal warts, sexual history and sexually transmitted diseases in PLWH. McCloskey et al. [22] identifies a strong increased risk of AIN with syphilis, genital herpes simplex virus and gonorrhoea infections as well as with increasing numbers of sexual partners it is possible that this is a hidden confounding factor.

In the past, ASCC has been associated with a higher incidence in women aged 60–70. More recently, clinical centres in England with high prevalence of HIV, such as Greater London, anecdotally have observed an increased frequency of younger male PLWH presenting with ASCC; this is demonstrated in the most recent ASCC incidence figures in England between 2013 and 2017. In England as a whole, there has been a 23% rise in incidence during this time period, with women having both a higher rate of incidence and larger increase in incidence rate. In contrast, in Greater London, although men still have a lower incidence rate as a whole than woman (1.41 cases per 100,000 people compared to 1.74 case per 100,000 people), men had a 50% increase in their incidence rate compared to only 5.5% in women in Greater London between 2013 and 2017 [23].

We anticipated that our clinical centre would have high numbers of male PLWH patients compared to the national average, but we were surprised that we had very low numbers of female patients with previous Genitourinary Intraepithelial Neoplasia (GIN) developing ASCC. This is unexpected, considering that the majority of patients with ASCC in England are female and it is likely that there is a significant cross over between HPV related dysplasia. There is a screening programme available for women with previous HPV related gynaecological dysplasia and this could be a sign that the programme is successful at our clinical centre, or it is possible that we have low rates of GIN in our region. If it is the latter, our results may not be generalisable to regions that have high volumes of patients with GIN.

A high number of our patients did not have a HIV test on clinical record. They were more likely to be female and have a previous diagnosis of Genitourinary Intraepithelial Neoplasia. It is possible that some of these patients may have had a negative HIV test elsewhere so a HIV test on diagnosis was not performed. It is very unlikely, that if patients tested positive for HIV elsewhere, for this diagnosis to be unknown to us. The untested patient subgroup was most statistically similar to the HIV negative patient subgroup (Table 3).

## Conclusion

We present a 20-year experience with ASCC in a HIV tertiary referral centre. We identified a patient subset of younger male PLWH developing ASCC. Patients known to have a previous diagnosis of AIN were more likely to have earlier disease.

There was no statistical difference in staging and disease-free survival and HIV status. However, PLWH were more likely to recur and when this occurred, it took longer to develop recurrent disease.

Five-year Disease-Free Survival was associated with tumour size and the absence of nodal or metastatic disease.

## Data Availability

The complete datasets generated and analysed in this study are not publicly available without ethical approval as they contain personally identifiable data from hospital databases. Anonymised supplementary information can be requested from the corresponding author if the request is reasonable and meets the conditions of the ethical approval granted to undertake this study. Not applicable.
